# Supplementary material for: First whole-genome detection of dengue virus in urban Aedes aegypti from Southern Brazil
Source: PLoS Negl Trop Dis. 2026 Mar 2;20(3):e0014050. doi: 10.1371/journal.pntd.0014050 (PMC12965692; doi:10.1371/journal.pntd.0014050)
Supplement: Attachment — Submitted filename: Comments for PNTD-D-25-01609.docx [file pntd.0014050.s003.docx]

This study presents the first whole-genome detection of dengue virus (DENV) from field-caught *Aedes aegypti* in southern Brazil, which is a notable and valuable contribution to the field of vector-borne disease surveillance. The manuscript is generally well-written, the methodology is sound, and the findings have clear relevance to public health. I believe the paper has the potential for publication after revisions.

Major Comments

Title and Abstract

1. The current title is accurate but somewhat lengthy. Consider shortening it for clarity and impact, e.g.,"First Whole-Genome Detection of Dengue Virus in Urban Aedes aegypti from Southern Brazil".
2. The abstract includes too much detail on the methodology. Please condense the methods section and emphasize the key findings and their implications, such as co-circulation of DENV-1 and DENV-2 and the potential for viral adaptation.

Introduction

1. While the background on dengue in Brazil is well covered, the manuscript would benefit from a broader international context regarding vector-based genomic surveillance. A few additional references from other dengue-endemic regions would help highlight the novelty and relevance of your approach.
2. Please clearly articulate the knowledge gap your study addresses, particularly the lack of genomic data from mosquitoes in southern Brazil.

Methods

1. The rationale for the selection of sampling sites should be better explained. Were these based on prior dengue cases, mosquito density, or ecological factors?
2. The experimental procedures are described in excessive detail. Please streamline the methodology, focusing on the key steps (e.g., RNA extraction, amplification, sequencing), and move less critical details to the supplementary materials.

Results

1. The identification of recurrent mutations in NS1, NS2A, and NS5 is interesting but underexplored. Please briefly discuss their potential functional relevance, even if speculative, based on existing literature.
2. The restricted geographic distribution of DENV-2 deserves a short explanation or hypothesis—could this be due to localized transmission chains, vector competence, or sampling bias?

Figures and Tables

1. Figure 1 is informative but could be improved:

Add a scale bar and legend for dot sizes/densities.

Improve color contrast for better visual accessibility.

2. Tables S2 and S3 contain valuable mutation data but are repetitive and hard to interpret. Consider merging them into a single summary table, grouping mutations by protein function, and indicating frequency of occurrence.

Discussion

1. The discussion would be strengthened by including a paragraph on study limitations, such as:

Cross-sectional design limiting temporal inference.

Pooling of mosquitoes preventing individual-level analysis.

Short sampling period (April–July) possibly missing seasonal dynamics.

2. Please also add future research directions, such as:

Longitudinal mosquito and human case surveillance.

Integration with climate and urbanization data.

Functional studies of identified mutations.
