# Supplementary material for: First whole-genome detection of dengue virus in urban Aedes aegypti from Southern Brazil
Source: PLoS Negl Trop Dis. 2026 Mar 2;20(3):e0014050. doi: 10.1371/journal.pntd.0014050 (PMC12965692; doi:10.1371/journal.pntd.0014050)
Supplement: Attachment — Submitted filename: Response to Reviewers.docx [file pntd.0014050.s004.docx]

Dear Editor,

We thank the reviewers for their time and contribution towards the review of our manuscript and for their encouraging remarks. We agree that the focus of the article is indeed the viral travel / historical movement in and out of Brazil have tried to emphasise this accordingly. We have addressed all comments made and we firmly believe that the revised manuscript has been greatly improved. We hope you might reconsider the current manuscript as suitable for publication in Plos Neglected Tropical Diseases.

**General comment:** This study presents the first whole-genome detection of dengue virus (DENV) from field-caught *Aedes aegypti* in southern Brazil, which is a notable and valuable contribution to the field of vector-borne disease surveillance. The manuscript is generally well-written, the methodology is sound, and the findings have clear relevance to public health. I believe the paper has the potential for publication after revisions.

**Reply:** We thank the reviewer for the positive and constructive comments. We appreciate the recognition of the novelty and public health relevance of our findings. We have revised the manuscript accordingly to address all suggestions and improve overall clarity.

**Journal Requirements:**

**1)** Please ensure that the CRediT author contributions listed for every co-author are completed accurately and in full. At this stage, the following Authors/Authors require contributions: Maria Eduarda Calazans Rodrigues, and Isaque João da Silva de Faria. Please ensure that the full contributions of each author are acknowledged in the "Add/Edit/Remove Authors" section of our submission form.

**Reply:** Done.

**2)** We do not publish any copyright or trademark symbols that usually accompany proprietary names, eg ©, ®, or TM (e.g. next to drug or reagent names). Therefore please remove all instances of trademark/copyright symbols throughout the text, including:

- ® on page: 4.

**Reply:** Done.

**3)** Please upload all main figures as separate Figure files in .tif or .eps format. For more information about how to convert and format your figure files please see our guidelines:

<https://journals.plos.org/plosntds/s/figures>

**Reply:** Done.

**4)** We notice that your supplementary figures are uploaded with the file type 'Table'. Please amend the file type to 'Supporting Information'. Please ensure that each Supporting Information file has a legend listed in the manuscript after the references list.

**Reply:** We thank the editorial team for the observation. We made the necessary changes.

**5) Potential Copyright Issues:**

Figure 1. Please (a) provide a direct link to the base layer of the map (i.e., the country or region border shape) and ensure this is also included in the figure legend; and (b) provide a link to the terms of use / license information for the base layer image or shapefile. We cannot publish proprietary or copyrighted maps (e.g. Google Maps, Mapquest) and the terms of use for your map base layer must be compatible with our CC BY 4.0 license.

**Reply:** For the base layer (administrative boundaries), we used the geobr R package, which downloads official Brazilian territorial boundary datasets (“Malhas territoriais”) from IBGE. Base layer source: geobr package (CRAN / project page) [https://cran.r-project.org/web/packages/geobr/geobr.pdf](https://cran.r-project.org/web/packages/geobr/geobr.pdf?utm_source=chatgpt.com) and IBGE Malhas Territoriais <https://www.ibge.gov.br/geociencias/organizacao-do-territorio/malhas-territoriais.html> . Terms of use / licensing information are available via IBGE’s open data page https://www.ibge.gov.br/acesso-informacao/dados-abertos.html.

**6)** Please amend your detailed Financial Disclosure statement. This is published with the article. It must therefore be completed in full sentences and contain the exact wording you wish to be published.

**Reply:** Done.

**Major Comments**

**Comment 1:** Title and Abstract - The current title is accurate but somewhat lengthy. Consider shortening it for clarity and impact, e.g.,"First Whole-Genome Detection of Dengue Virus in Urban Aedes aegypti from Southern Brazil".

**Reply:** Done.

**Comment 2:** The abstract includes too much detail on the methodology. Please condense the methods section and emphasize the key findings and their implications, such as co-circulation of DENV-1 and DENV-2 and the potential for viral adaptation.

**Reply:** We thank the reviewer for this valuable suggestion. The abstract has been revised to substantially condense the methodological details and to emphasize the main findings and their implications. Specifically, we reduced descriptions related to sampling strategy and sequencing workflow, while highlighting the co-circulation of DENV-1 and DENV-2, evidence of simultaneous circulation in mosquito pools, and genomic signals consistent with ongoing viral adaptation. These changes improve clarity and better align the abstract with the study’s primary contributions.

**Comment 3:** Abstract: The aim should be made clear.

**Reply:** Done.

**Comment 4:** Introduction - The aim is not clear; it should be made clear. Is the aim mosquito-trapping or quantify the circulating dengue virus serotypes.

**Reply:** We thank the reviewer for this comment. The Introduction has been revised to clearly state the study aim. We now explicitly define the objective as characterizing circulating dengue virus serotypes and their genomic features in southern Brazil using vector-based surveillance, rather than focusing on mosquito trapping as the primary aim. This clarification better aligns the Introduction with the study’s scope and findings.

**Comment 5:** The study - The process of sampling is very detailed, summarize the process. - Limitation of study is needed.

**Reply:** We thank the reviewer for bringing this to our attention. We have made the necessary edits.

**Comment 6:** Introduction: While the background on dengue in Brazil is well covered, the manuscript would benefit from a broader international context regarding vector-based genomic surveillance. A few additional references from other dengue-endemic regions would help highlight the novelty and relevance of your approach.

**Reply:** We thank the reviewer for this suggestion. We have revised the Introduction to place our study within a broader international context by highlighting previous vector-based genomic surveillance efforts conducted in other dengue-endemic regions, including Southeast Asia and Central America. These additions help contextualize our work globally and emphasize the novelty and relevance of applying vector-based genomic surveillance in southern Brazil.

**Comment 7:** Please clearly articulate the knowledge gap your study addresses, particularly the lack of genomic data from mosquitoes in southern Brazil.

**Reply:** We thank the reviewer for this comment. The Introduction has been revised to more clearly articulate the knowledge gap addressed by this study, specifically the lack of vector-derived genomic data for dengue virus in southern Brazil. We now explicitly highlight how this gap limits understanding of serotype circulation and viral evolution in a region undergoing rapid epidemiological transition.

**Reviewers' Comments:**

**Comment 8:** Methods: The rationale for the selection of sampling sites should be better explained. Were these based on prior dengue cases, mosquito density, or ecological factors?

**Reply:** Our sampling strategy prioritized ecological and socio-spatial diversity and was optimized to detect vectors in urban zones typically underrepresented in entomological surveillance.

**Comment 9:** The experimental procedures are described in excessive detail. Please streamline the methodology, focusing on the key steps (e.g., RNA extraction, amplification, sequencing), and move less critical details to the supplementary materials.

**Reply:** We thank the reviewer for the suggestion. The Methods section has been streamlined to focus on the main experimental steps, and less essential details were moved to the Supplementary Materials.

**Comment 10:** The manuscript demonstrated the whole-genome evidence of dengue virus in field-caught mosquitoes from southern Brazil. The method is precise; however, some issues need to be clarified. **Results:** The identification of recurrent mutations in NS1, NS2A, and NS5 is interesting but underexplored. Please briefly discuss their potential functional relevance, even if speculative, based on existing literature.

**Reply:** We thank the reviewer for this insightful comment. The Discussion has been revised to briefly address the potential functional relevance of recurrent mutations detected in NS1, NS2A, and NS5, drawing on existing literature regarding their roles in viral replication and immune interaction. While avoiding overinterpretation, we now highlight that the recurrent involvement of these non-structural proteins may reflect selective pressures acting on dengue viruses circulating in expanding transmission zones, and we explicitly note that functional validation will be required to determine the biological impact of these substitutions.

**Comment 11: The restricted geographic distribution of DENV-2 deserves a short explanation or hypothesis—could this be due to localized transmission chains, vector competence, or sampling bias?**

**Reply:** We thank the reviewer for this important observation. We now briefly acknowledge in the Discussion that the restricted geographic distribution of DENV-2 observed in this study may reflect localized transmission chains occurring within a broader epidemiological context dominated by other serotypes, particularly DENV-1 during the study period. In addition, we note that uneven sampling intensity across locations may have contributed to this pattern and cannot be excluded as a source of bias. While differences in vector competence represent another possible explanation, the available data do not allow us to robustly assess this hypothesis. We therefore frame the observed distribution as the likely result of localized viral circulation and sampling heterogeneity rather than as evidence of a strict biological constraint, and highlight the need for broader and more systematic surveillance to disentangle these factors.

**Comment 12:** Figures and Tables: Figure 1 is informative but could be improved:

a. Add a scale bar and legend for dot sizes/densities.
b. Improve color contrast for better visual accessibility.

**Reply:** We thank the reviewer for the constructive suggestions regarding Figure 1. We would like to clarify that the purpose of this figure is to illustrate the geographic location of mosquito sampling sites rather than to represent sampling density or quantitative intensity. For this reason, dot size was kept uniform and no density legend was included. Regarding color contrast, the figure was designed using color palettes selected to ensure visual clarity and accessibility, including consideration for color-vision deficiencies, following established guidelines and tools commonly used in scientific visualization (e.g., ColorBrewer and similar scientific color selection frameworks). We therefore believe that the current version of Figure 1 appropriately conveys the intended information and maintains adequate visual accessibility without further modification.

**Comment 13:** Figures and Tables: Tables S2 and S3 contain valuable mutation data but are repetitive and hard to interpret. Consider merging them into a single summary table, grouping mutations by protein function, and indicating frequency of occurrence.

**Reply:** We thank the reviewer for the suggestion. Tables S2 and S3 were initially kept separate to preserve transparency and detailed reporting of mutation data, but they have now been merged (Table S2) in response to the reviewer’s request, and the suggested modifications have been implemented.

**Comment 14:** The figure 1, is not informative and very busy to comprehend. The authors should reconstruct the phylogenetic tree with the concise needed information (fewer reference sequences from the Genbank) and outgroup. The authors should also describe each clade clearly for the readers.

**Reply:** We thank the reviewer for this comment. In accordance with the journal’s guidelines for Short Reports, the manuscript is limited to a single main figure, which was therefore designed to integrate both the phylogenetic context and the spatial information necessary to support the study objectives. To simplify the datasets while retaining representative sequences and improving visualization, we grouped sequences by collection location, week, and year, removed redundant sequences with CD-Hit, and excluded outliers identified through TreeTime analyses. This reduced the Dengue 1 dataset from 5,354 to 1,628 sequences and the Dengue 2 dataset from 3,614 to 1,492 sequences. To maintain consistency with previous dengue virus phylogenetic studies and given the well-established evolutionary relationships among DENV lineages, no distant outgroup was included, as this would not add meaningful resolution to the analysis. The final figure thus maintains clarity and visual coherence, allowing readers to readily interpret both phylogenetic and spatial patterns in a manner consistent with prior studies.

**Comment 15:** Discussion: The discussion would be strengthened by including a paragraph on study limitations, such as:

a. Cross-sectional design limiting temporal inference.
b. Pooling of mosquitoes preventing individual-level analysis.
c. Short sampling period (April–July) possibly missing seasonal dynamics.

**Reply:** We thank the reviewer for this suggestion. In the revised manuscript, we have added a dedicated Limitations section explicitly addressing the main constraints of the study. These include the cross-sectional design, which limits temporal inference; the use of pooled mosquito samples, which prevents individual-level infection analysis and may reduce sensitivity for low-prevalence infections; and the restricted four-month sampling period (April–July), which may not capture seasonal variation in dengue virus circulation.

**Comment 16:** Please also add future research directions, such as:

d. Longitudinal mosquito and human case surveillance.
e. Integration with climate and urbanization data.
f. Functional studies of identified mutations.

**Reply:** We appreciate this constructive comment. Future research directions have now been incorporated into the Discussion, highlighting the need for longitudinal mosquito-based and human case surveillance to better capture temporal dynamics. We also emphasize the importance of integrating genomic data with climatic and urbanization variables to refine risk assessment and transmission modeling. In addition, we point to the value of functional studies to investigate the biological relevance of the identified mutations and their potential impact on viral fitness, transmission, and immune interactions.

**Comment 17:** The manuscript currently does not include a clearly defined Discussion section. Adding this section would help contextualize the findings and relate them to existing literature. The manuscript would also benefit from the inclusion of a brief statement addressing the limitations of the study. Highlighting these limitations will provide a more balanced interpretation of the results and strengthen the overall presentation.

**Reply:** We appreciate the reviewer’s suggestion. In the revised manuscript, we added a dedicated Discussion section to better place our findings in the context of existing literature. This section discusses the relevance of our results for dengue virus genomic surveillance and vector-based detection. We also included a brief statement describing the main limitations of the study, including the sample size and the limited temporal and geographic scope of mosquito collections. We believe that addressing these points helps provide a more balanced interpretation of the results and improves the overall clarity of the manuscript.

**Comment 18:** Editorial and Data Presentation Modifications: In the Background section, it would be helpful to include additional general information about the virus, such as the approximate size of its genome.

**Reply:** We thank the reviewer for this suggestion. In the revised Background section, we have added general information about the virus, including the approximate size of its genome. Specifically, we now state that the DENV genome is approximately 10.7 kb in length and encodes three structural proteins (C, prM/M, and E) and seven nonstructural proteins (NS1–NS5).

**Comment 19:** The abbreviation “NS” should be formally defined at first mention.

**Reply:** The abbreviation “NS” refers to nonstructural proteins of the dengue virus and has now been formally defined at its first mention in the manuscript.

**Comment 20:** The manuscript presents genomes sequenced from specimens collected in 2023 and shown on a phylogenetic tree spanning isolates from 1991 to 2023 (Figure 1B). However, the compressed time scale makes it appear that the 2023 genomes are closer to the August 2021 time point, which may cause confusion. It would be useful to clearly mark or label the 2023 point on the timeline.

**Reply:** We thank the reviewer for bringing this to our attention. We have now made the necessary changes.

**Comment 21:** The formatting of the Supplemental Tables needs correction. Several tables contain truncated text, overlapping content across pages, and columns that are too narrow—causing numerical values to appear as “###.” Please adjust the layout to ensure all text and data are clearly visible.

**Reply:** Done.

**Comment 22:** The manuscript aims to address an important knowledge gap regarding DENV strains circulating in underrepresented regions of Brazil. While the methods used for genomic sequencing appear generally adequate, the methodological details are interspersed with the results and discussion. This formatting choice makes it difficult to clearly and critically evaluate the methods. The authors are encouraged to present the Methods section separately to improve clarity and reproducibility.

**Reply:** We thank the reviewer for the comment and for highlighting the importance of methodological clarity. For Short Reports, the journal’s guidelines do not allow a standalone Methods section, and methodological details are therefore presented alongside the study description. In addition, the editorial assessment explicitly requested a shortened version of the methods, which we have followed meticulously.

**Comment 23:** Line 117: The authors state that “outliers were removed to improve dating accuracy.” Please provide additional detail on the parameters or criteria used to identify and remove these outliers, as this information is essential for evaluating the validity of the temporal analysis

**Reply:** We thank the reviewer for this comment. Outliers were identified and removed based on standard molecular clock diagnostics, specifically by assessing root-to-tip regression and temporal signal consistency. Sequences showing clear deviations from the expected linear relationship between genetic divergence and sampling time were excluded to improve dating reliability. This approach follows commonly used practices in phylodynamic analyses and aims to reduce noise introduced by sequences with potential sequencing errors, incorrect sampling dates, or atypical evolutionary rates.

**Comment 24:** Summary and General Comment: The authors should provide citations to their statements below:

- **Line 58-59 “Environmental and anthropogenic factors……….”**

**Reply:** Done.

- **Line 75-76 “ In 2023, 38,176 cases………”**

**Reply:** Done

**Comment 25:** The authors mentioned they pooled both males and females into tubes. What is the essence of including males in the study?

**Reply:** We included both male and female *Aedes aegypti* in the pooled samples because males can occasionally harbor dengue virus through vertical (transovarial) transmission.

**Comment 26:** How long did the authors preserved the samples without significant RNA degradation and what is the medium of preservation (solution?)?

**Reply:** The mosquito samples were preserved immediately after collection in a guanidinium thiocyanate–based lysis buffer, which stabilizes nucleic acids and prevents RNA degradation. All samples were processed within 7 days of collection, a timeframe in which RNA integrity is reliably maintained under this preservation method.

**Comment 27:** Line 103: typo “for” “for” delete one.

**Reply:** Done.

**Comment 28:** Regarding the RNA extraction, it can be found that the genomic DNA, if not digested, can be present in the eluted RNA, resulting possible positive amplification of the gene of interest in tested specimens. Did the authors decontaminate the DNA before amplification?

**Reply:** Thank you for this important point. We used the Bio Gene Viral DNA/RNA Extraction Kit (K204), which performs guanidine-based lysis and silica-column purification and co-purifies viral DNA and RNA as described in the manufacturer’s manual. The standard kit protocol does not include a DNase step. Importantly, our diagnostic workflow relied on RT-dependent assays (one-step RT-qPCR and RT-PCR), and primers/probes used are specific to dengue virus genomic RNA sequences that are absent from the mosquito genome. Therefore, genomic DNA from mosquitoes is not expected to serve as an amplification template for the viral assays. To reduce the risk of false positives due to contamination, all runs included no-template controls and standard laboratory contamination controls.

**Comment 29:** The authors should provide the serotype-specific primers used in the study?

**Reply:** Thank you for this comment. In this study, we used serotype-specific primer schemes developed by the CADDE project (https://www.caddecentre.org/) for whole-genome sequencing of DENV-1 and DENV-2. These primers are publicly available and were designed for multiplex PCR amplification of complete viral genomes. We have revised the Methods section to clarify this point and added the appropriate references.

**Comment 30:** Could the authors clarify the pooling strategy used in this study, given that many tubes contained single (individual) samples despite being referred to as pools, while Table S1 shows unequal pool sizes ranging from single specimens to pools containing 2–15 flies per tube; additionally, please specify the sex of individuals in single-sample tubes and provide the total number of male and female flies included in the study?

**Reply:** Thank you for this observation. In this study, mosquito pools were constructed per trap and per week, meaning that the number of mosquitoes in each pool depended on the capture yield of a given trap during a specific sampling interval. As a result, some traps captured only a single mosquito, leading to pools containing one individual. These single-mosquito pools were therefore not methodological exceptions but rather reflect natural variability in trap captures. When a pool contained only one mosquito, the individual was always a female, whereas males were present only in multi-mosquito pools. To improve clarity, we will revise the Methods section to specify that pools were constructed according to trap location and collection date, and that pool size varied depending on trap yield, including single-mosquito pools representing traps that captured only one individual during that week.

**Comment 31:** The 2 pools the author mentioned that were positive for both DENV I and 2, how many samples in each pool? All males or females?

**Reply:** Thank you for your questions. In total, the study included predominantly female *Aedes aegypti*, and none of the DENV-positive pools contained male mosquitoes. Specifically, the two pools that were positive for both DENV-1 and DENV-2 (pools 23 and 28) each contained only female mosquitoes, with no males present in their composition.

**Comment 32:** The authors claimed that they had 33 positive samples out of 41 pools for DENV: 29 for DENV-1 and 4 for DENV-2. How accurate is this high infection rate? Does the author understand the epidemiological implication of this claim. The chance of possible transmission is very high in the study areas. These results are discussable.

**Reply:** Thank you for raising this important point. To avoid misinterpretation of the infection rate, we have now added the total number of pools analyzed in the revised manuscript. This makes it clear that the 41 DENV-positive pools represent only a small fraction of all 2,022 pools screened, and therefore do not reflect a high infection rate in the mosquito population. The detection of 33 genomes from these 41 positive pools reflects successful sequencing, not a high prevalence of infection in the field. We clarified this in both the Methods and Results to prevent any misunderstanding regarding the epidemiological implications.

**Comment 33:** How did the authors avoid contaminations amongst sample analyzed?

**Reply:** Regarding contamination control, we followed strict laboratory practices to avoid cross-contamination. Each batch of RNA extraction, RT-qPCR, amplification, and sequencing included positive controls (validated DENV RNA) and negative extraction controls to identify any potential contamination during the process. No amplification or sequencing signal was observed in negative controls at any point in the workflow, indicating that contamination did not occur. Additionally, RNA extraction, PCR setup, and post-PCR processing were conducted in physically separated laboratory areas using filtered tips and dedicated equipment, following standard procedures for arbovirus molecular diagnostics.

**Comment 34:** Line 127: Were the CT values analyzed by the comparative threshold cycle method, and normalized to the endogenous/reference control?

**Reply:** Thank you for this question. Our objective was to determine the presence or absence of DENV in mosquito pools. For this purpose, Ct interpretation was based solely on specific amplification of the viral target, together with positive and negative controls, which is the standard approach for arbovirus surveillance in mosquitoes. Because DENV is an RNA virus and the target region is absent from the mosquito genome, normalization to an endogenous gene is not required and does not influence diagnostic classification. Importantly, extraction controls were included to confirm that the RNA extraction and amplification steps functioned as expected, and no amplification was observed in negative controls throughout the workflow.

**Comment 35:** Line 135 -140: Not clearly demonstrated in the Figure 1B-C, very difficult to follow.

**Reply:** We thank the reviewer for the comment. The statements in lines 135–140 are supported by the phylogenetic structure and geographic annotation shown in Figure 1B–C. In Figure 1B, DENV-1 sequences generated in this study (highlighted in red) are distributed across four distinct clades within genotype V, with several clustering predominantly with sequences from southern Brazil, consistent with ongoing local transmission, while others group with sequences from different geographic regions, indicating multiple introduction events. In Figure 1C, DENV-2 sequences from this study are nested within previously sampled southern Brazilian lineages of genotype II, preceding the 2024 outbreak and supporting their regional origin. Given the Short Report format and the need to present all phylogenetic results within a single figure, we consider the current visualization and its correspondence with the text appropriate.

**Comment 36:** Overall, the observed results needs more discussion.

**Reply:** Thank you for this comment. The Discussion section has been expanded to better interpret the observed results and place them in the context of existing literature.

**Comment 37:** There could be substantial additional value to the findings reported in this manuscript if the authors extended their analysis. While they allude to the importance of understanding the impact of the variants identified in the sequenced DENV genomes, they do not perform the bioinformatic analyses necessary to address this knowledge gap. With the availability of advanced structural modeling tools (e.g., AlphaFold3), it would be feasible to assess the potential structural or functional implications of the identified variants using a reference DENV strain as a model template. Including such analyses would considerably strengthen the study’s impact and interpretive depth.

**Reply:** We appreciate the reviewer’s suggestion and agree that structural or functional analyses of the identified variants could provide additional biological insight. However, the main aim of this study was to report the detection of complete DENV genomes and to describe their genomic diversity in field-caught mosquitoes, as part of vector-based genomic surveillance. Detailed proteomic or structural modeling analyses were therefore beyond the scope of this work and would require a separate, more targeted investigation. We have acknowledged this limitation and indicated that future studies could further explore the functional implications of the variants identified here.
