# Supplementary material for: First whole-genome detection of dengue virus in urban Aedes aegypti from Southern Brazil
Source: PLoS Negl Trop Dis. 2026 Mar 2;20(3):e0014050. doi: 10.1371/journal.pntd.0014050 (PMC12965692; doi:10.1371/journal.pntd.0014050)
Supplement: Attachment — Submitted filename: Response to Reviewers 2.docx [file pntd.0014050.s005.docx]

**Additional Editor Comments:** One reviewer has identified **some minor changes** that should be addressed prior to formal acceptance. Please note that PLOS journals do not edit manuscripts once they are accepted so it is the authors responsibility to ensure grammar, readability and, in this case, figure layout are publication quality. Once these modifications are complete, this manuscript should be suitable for acceptance at the editor level. -MH

**Reply:** Thank you for the reviewer’s helpful comments. We have carefully revised the manuscript to address the suggested minor changes, including improvements to grammar, readability, and figure layout, and we believe it is now suitable for acceptance at the editor’s level.

**Journal Requirements:**

1) Please ensure that the CRediT author contributions listed for every co-author are completed accurately and in full. At this stage, the following Authors/Authors require contributions: **Maria Eduarda Calazans Rodrigues**. Please ensure that the full contributions of each author are acknowledged in the "Add/Edit/Remove Authors" section of our submission form.
The list of CRediT author contributions may be found here: <https://journals.plos.org/plosntds/s/authorship#loc-author-contributions>

**Reply:** The CRediT contributions for Maria Eduarda Calazans Rodrigues (MECR) have been completed and include roles in investigation and revision, as reflected in the submission form.

2) Please upload all main figures as separate Figure files in .tif or .eps format. For more information about how to convert and format your figure files please see our guidelines:
<https://journals.plos.org/plosntds/s/figures>

**Reply:** Done.

3) Tables should not be uploaded as individual files. Please remove these files and include the Tables in your manuscript file as editable, cell-based objects. For more information about how to format tables, see our guidelines: https://journals.plos.org/plosntds/s/tables

**Reply:** The tables referred to are supplementary tables (Supporting Information) and therefore were not included in the main body of the manuscript. They have been labeled as S1 Table and S2 Table and provided as separate supporting information files in accordance with PLOS guidelines.

4) Please amend your detailed Financial Disclosure statement. This is published with the article. It must therefore be completed in full sentences and contain the exact wording you wish to be published.
- State the initials, alongside each funding source, of each author to receive each grant. For example: "This work was supported by the National Institutes of Health (####### to AM; ###### to CJ) and the National Science Foundation (###### to AM)."

- State what role the funders took in the study. If the funders had no role in your study, please state: "The funders had no role in study design, data collection and analysis, decision to publish, or preparation of the manuscript.". If you did not receive any funding for this study, please simply state: u201cThe authors received no specific funding for this work.u201d

**Reply**: The Financial Disclosure statement has been amended and rewritten in full sentences, using the exact wording intended for publication. The revised statement now clearly identifies the funding source and specifies the role of the funder in the study, in accordance with the journal’s guidelines.

**Reviewers' comments:**

**Methods**

**Reviewer #1:** The objectives of the study clearly articulated with a clear testable hypothesis stated

**Reply:** We thank the reviewer for the positive comment.

**Editorial and Data Presentation Modifications?**

**Reviewer #1:** Having reviewed the authors' responses to my previous concerns, I find that all issues have been satisfactorily addressed. I now recommend that the manuscript be accepted for publication.

**Reply:** We thank the reviewer for the positive evaluation and for confirming that all concerns have been satisfactorily addressed. We appreciate the recommendation for acceptance of our manuscript.

**Summary and General Comments**

**Reviewer #3:** The manuscript has improved considerably with the current revision. The authors have done a commendable job expanding the discussion and acknowledging the study’s shortcomings through the addition of a limitations section. However, a few minor issues remain that should be addressed prior to acceptance.

**Reply:** We thank the Reviewer for the positive and constructive evaluation of the revised manuscript. All remaining minor comments have been carefully addressed in the current version of the manuscript, and we believe that these revisions have further strengthened the clarity and quality of the work.

**Comment 1**: On lines 155–157, the authors state that the distributions of DENV-1 and DENV-2 occur in different geographic locations, as shown in Figure 1A. However, all data points appear in the same color, preventing the reader from distinguishing between DENV-1 and DENV-2 by region as suggested in the text.

**Reply:** The figure has been revised accordingly to clearly distinguish between DENV-1 and DENV-2 by region.

**Comment 2**: Additionally, many values in Supplemental Table 1 are still displayed as “####” in the CT values, total reads, and Deep columns. The authors indicated that this issue was corrected during revision, but it does not appear to have been fully resolved.

**Reply:** We thank the reviewer for the comment. The issue was caused by the decimal separator format and has now been corrected. All values in Supplemental Table 1 are displayed correctly.

**Comment 3**: Finally, there are multiple instances throughout the manuscript of extra spaces and an occasional additional period. The manuscript would benefit from another careful proofreading to address these minor typographical issues and to ensure consistent use of abbreviations throughout.

**Reply:** We thank the reviewer for this observation. The manuscript has been carefully proofread, and all identified instances of extra spaces and additional punctuation have been corrected.
